# Proteomics-Based Analysis of Structural Changes and Functional Properties of Casein Derived from Qula

**DOI:** 10.3390/foods15203669

**Published:** 2026-10-15

**Authors:** Ting Mao, Yongqiang Huang, Jinyong Ma, Jinwu Yan, Juan Qin, Zhiye Wang

**Affiliations:** 1 Key Laboratory of Microbial Resources Exploitation and Application of Gansu Province, Institute of Biology, Gansu Academy of Science, Lanzhou 730000, China; maotingjiayou@126.com; 2 Jinhui Liquor Co., Ltd., Longnan 742300, China; hyq@jinhuijiu.com (Y.H.); yjw@jinhuijiu.com (J.Y.); 3 Gansu Puluo Biotechnology Co., Ltd., Yumen 735200, China; hxmjy@163.com

**Keywords:** Qula, casein, proteomics, protein structure, functional properties analysis

## Abstract

To elucidate the molecular basis of the functional deterioration of Qula-derived casein, proteomic analysis was conducted, and the protein composition, structural characteristics, and functional properties of imported casein, fresh yak milk casein, and Qula-derived casein were systematically compared. Structural characterization was performed by spectroscopic and microscopic analyses, and physicochemical properties were measured in parallel. Qula-derived casein contained higher proportions of αs1-casein than imported casein and β-casein than both imported and fresh yak milk casein, but a lower proportion of κ-casein than either sample. Relative to fresh yak milk casein, Qula-derived casein showed decreases of 31.8% and 12.3% in α-helix and β-sheet contents, respectively, accompanied by a 22.3% increase in random coil content. Its microstructure also changed from a loose, porous morphology to dense, block-like aggregates. Accordingly, its emulsifying activity index, emulsion stability index, solubility, and surface hydrophobicity decreased to 29.3 m^2^/g, 23 min, 0.6%, and 199, respectively, representing reductions of 36.7%, 25.8%, 33.3%, and 32.1% compared with fresh yak milk casein. These findings indicate that alterations in casein isoform composition are associated with protein unfolding, aggregation, and the deterioration of functional properties during Qula processing. The new knowledge of this study lies in providing insight into the molecular mechanism by which traditional Qula processing may induce casein subtype changes, unfolding, and aggregation, and in describing a possible association between decreased κ-casein abundance and impaired functionality. These findings support the value-added utilization of Qula-derived casein and the optimization of traditional processing.

## 1. Introduction

Gansu Province has substantial potential for dairy-industry development; however, limited collection and processing capacity results in considerable losses of fresh milk. To preserve surplus milk and prevent spoilage, local herders traditionally process yak milk into butter and Qula. Qula is a dried dairy product prepared from skimmed yak milk through acid coagulation, heating, and natural air-drying [1]. It consists predominantly of casein, with only minor residual amounts of milk fat and lactose [2]. Although Qula offers economic and resource-related advantages as a raw material for casein production, prolonged air-drying promotes protein aggregation and denaturation, thereby impairing its functional properties relative to casein obtained from fresh milk. These limitations restrict the application of Qula-derived casein in value-added food products [3].

Casein is the predominant milk phosphoprotein, accounting for approximately 80% of total milk protein. Industrial casein is typically produced by acid- or enzyme-induced precipitation of skim milk, followed by purification and drying. Heat treatment induces diverse structural modifications in milk proteins, including Maillard reactions, methionine oxidation, and covalent cross-linking and aggregation. Li et al. [4] reported that β-lactoglobulin undergoes thiol–disulfide exchange and disulfide-bond rearrangement at 60–90 °C, while Lowe et al. [5] confirmed the formation of covalent β-lactoglobulin–κ-casein complexes through the same mechanism during heating. Gao et al. [6] further demonstrated that heating promotes protein structural modification and aggregation, as indicated by increased levels of soluble aggregates. Fourier transform infrared spectroscopy revealed substantial changes in the secondary structure of casein after ultrahigh-temperature treatment, characterized by decreases in α-helix and β-sheet contents and an increase in β-turn content [7]. These heat-induced changes can alter casein micelle morphology and molecular flexibility, thereby affecting its functional properties [8]. Moreover, sodium caseinates from different sources exhibit significant structural and functional differences, with a higher κ-casein content being associated with improved emulsifying performance [9]. These structural changes affect functional properties because the emulsifying, solubility, and gelling properties of casein depend on its micelle structure, surface hydrophobicity, molecular flexibility, and aggregation state, with surface-located κ-casein being particularly critical and its decreased content generally associated with impaired emulsifying performance.

Most previous studies have examined casein derived from fresh milk, whereas systematic investigations of the proteomic profile, structural characteristics, and functional properties of casein prepared from Qula-a traditional dairy product subjected to natural fermentation, thermal treatment, and prolonged sun-drying-remain scarce [10]. Owing to its distinctive processing history, Qula-derived casein may possess an initial conformation that differs substantially from that of casein obtained from conventional milk sources. Elucidating the mechanisms underlying structural and functional alterations is therefore essential for the value-added utilization of Qula [11]. Proteomic analysis provides a powerful approach for characterizing changes in protein composition and post-translational modifications at the molecular level, thereby facilitating the identification of their contributions to casein functionality [12].

In this study, Qula-derived casein and fresh yak milk casein were both obtained from yak milk, while imported casein was used as an industrial reference control to systematically compare the proteomic profiles, secondary structures, microstructures, and functional properties of Qula-derived casein. The objective was to elucidate the molecular mechanisms underlying its functional deterioration and provide a theoretical basis for its application potential in food products.

## 2. Materials and Methods

### 2.1. Sample Preparation

The raw milk used for Qula preparation was yak milk. Yak milk is a casein-type milk, with casein accounting for approximately 80% of total protein and whey proteins for approximately 20%. Its casein consists of αs1-, αs2-, β-, and κ-casein, among which αs1-, αs2-, and β-casein are located predominantly in the micelle interior, whereas κ-casein is mainly on the micelle surface and serves as a stabilizer. Compared with cow milk, yak milk has a higher proportion of β-casein, exceeding 45%, and a slightly lower proportion of κ-casein, approximately 15% [13]. The pH of yak milk ranges from 6.36 to 6.62, and its titratable acidity is approximately 23.04 °T [14]. The protein, fat, and lactose contents are approximately 4.12%, 5.69%, and 4.71%, respectively [15].

Sample A (imported casein) was purchased from Fonterra Co-operative Group Ltd. (Auckland, New Zealand). The supplier did not provide species-specific information; therefore, Sample A was used only as industrial reference control. Sample B (fresh yak milk casein) was prepared from yak milk. Briefly, milk was centrifuged at 3000 rpm for 20 min using a centrifuge (Hunan Xiangyi Laboratory Instrument Development Co., Ltd., Changsha, China) to remove fat. The skim milk was acidified to pH 4.6 with 1 mol/L HCl using a pH meter (Shanghai INESA Scientific Instrument Co., Ltd., Shanghai, China) and held for 2 h to induce casein precipitation [16]. The precipitate was collected by centrifugation, washed three times with deionized water at 30 °C, and spray-dried. Sample C (Qula-derived casein) was prepared from Qula, a traditional dairy product made from yak milk.

Qula is a traditional handmade dairy product, and its processing conditions vary among herders and batches. Based on the local traditional practice, yak milk is defatted and then subjected to natural fermentation for approximately 24–48 h, followed by heating at about 50–60 °C for 20–40 min to expel whey, and finally air-drying under natural sunlight. The Qula raw material was collected from Gannan Tibetan Autonomous Prefecture, Gansu Province, China, and provided by Gansu Puluo Biotechnology Co., Ltd. (Yumen, Gansu, China), representing the local traditional processing practice. The three biological replicates were obtained from three independent batches, and batch-to-batch variation in protein content and color was observed. Briefly, 500 g of Qula was accurately weighed using an analytical balance (Sartorius, Göttingen, Germany) and dispersed in water at a mass ratio of 1:3 and stirred at 300 rpm until homogeneous [17]. The dispersion was centrifuged sequentially at 4000–6000 rpm for 20 min and 6000–8000 rpm for 20 min. The collected supernatant was gradually acidified to pH 4.6 with 3 mol/L HCl and held for 2 h to ensure complete protein precipitation [18]. The precipitate was washed three times with 100 mL of deionized water at 30 °C, redispersed in an appropriate volume of deionized water, and spray-dried using a BILON laboratory spray dryer (Shanghai Bilang Instrument Manufacturing Co., Ltd., Shanghai, China).

The prepared casein samples were analyzed according to the National Food Safety Standard for Casein (GB 31638-2016) [19]. Protein content was determined by the Kjeldahl method (GB 5009.5-2016) using Kjeldahl apparatus (Jinan Hanon Instruments Co., Ltd., Jinan, China). [20]. Protein content was calculated by multiplying the total nitrogen content by a conversion factor of 6.38, fat content by Soxhlet extraction (GB 5009.6-2016) [21], moisture content by the direct drying method (GB 5009.3–2016) [22], and ash content by high-temperature incineration (GB 5009.4-2016) [23]. Ash content was determined as an estimate of the total mineral content of the samples.

### 2.2. Proteomic Analysis

The proteomic analysis was performed by Biomarker Technologies Co., Ltd. (Beijing, China), and all reagents used in this section were provided by this company. Three biological replicates were analyzed for each of the three casein samples. Each biological replicate was independently prepared from a separate batch of raw material. Samples were suspended in lysis buffer containing 8 mol/L urea and 50 mmol/L Tris–HCl (pH 8.0), and protein concentrations were determined. Equal amounts of protein were reduced with 10 mmol/L dithiothreitol at 56 °C for 30 min and alkylated with 20 mmol/L iodoacetamide at room temperature for 30 min in the dark. The proteins were digested overnight at 37 °C with trypsin at a protein-to-enzyme ratio of 50:1 (*w*/*w*). Peptides were desalted using C18 solid-phase extraction cartridges, vacuum-dried, and redissolved in 0.1% formic acid.

Peptide mixtures were analyzed by nanoflow liquid chromatography–tandem mass spectrometry (nanoLC–MS/MS). The mobile phases were solvent A (0.1% formic acid in water) and solvent B (0.1% formic acid in acetonitrile) [24]. Peptides were separated using gradient elution and analyzed on an Orbitrap Astral mass spectrometer (Thermo Fisher Scientific, Waltham, MA, USA) operated in data-independent acquisition (DIA) mode [25]. Raw DIA data were processed using DIA-NN software (version 1.9.2). The raw data were searched against the UniProt Bos taurus reference proteome (Proteome ID UP000009136, downloaded on 18 August 2024). Although the Qula-derived casein and fresh yak milk casein samples were derived from yak milk, yak and cattle belong to the same family Bovidae and share highly conserved protein sequences, and the Bos taurus reference proteome is commonly used for cross-species proteomic analysis of yak milk [26,27].

The number of entries in the UniProt yak reference proteome is limited. Because the main conclusions focus on the relative abundances of the four highly conserved casein subtypes in the yak milk samples (Samples B and C), this database choice is unlikely to affect the main relative abundance comparisons. A spectral library was predicted using the deep learning algorithm in DIA-NN and combined with the library obtained by the match-between-runs (MBR) function to extract protein quantification information from the DIA raw data. Missed cleavages were set to 2, trypsin was used as the digestion enzyme, and the final results were filtered at a 1% FDR at both the precursor and protein levels. A total of 7264 unique peptides were identified across all samples. Proteins with more than 50% missing values in any single group were removed, and remaining missing values were imputed using the k-nearest neighbors (KNN) algorithm.

Label-free quantification was performed, and protein abundances were normalized to the total peak area of each sample [28]. Differentially abundant proteins (DAPs) were identified using the criteria of a fold change (FC) ≥ 2 and *p* < 0.05 [29]. The *p*-values used for DAP identification were raw *p*-values without multiple-testing correction. This analysis was exploratory and intended to screen candidate DAPs rather than to perform confirmatory statistical inference; the FC ≥ 2 threshold provided additional filtering. SDS-PAGE results showing clear main bands for αs1/αs2-casein, β-casein, and κ-casein in all three samples are provided in Supplementary Figure S1.

### 2.3. Fourier Transform Infrared Spectroscopy Analysis

The secondary structures of the three casein products were characterized using a Thermo Scientific Nicolet iS50 FTIR spectrometer (Thermo Fisher Scientific, Madison, WI, USA), as previously described [30]. Briefly, 2 mg of each casein sample was accurately weighed and thoroughly mixed with 200 mg of dried spectroscopic-grade potassium bromide (KBr) powder (Servicebio Technology Co., Ltd., Wuhan, China) in an agate mortar. The homogeneous mixture was compressed into a transparent pellet using a pellet die under a pressure of 10 t/cm^2^ for 1–2 min.

A pure KBr pellet was used as background reference, and the sample spectra was collected immediately after pellet preparation [31]. FTIR spectra were recorded over the range of 4000–500 cm^−1^ at a resolution of 4 cm^−1^, with 32 scans accumulated for each sample. The obtained spectra were processed using OMNIC software (version 9.8; Thermo Fisher Scientific, Waltham, MA, USA), including baseline correction and smoothing.

### 2.4. Circular Dichroism Spectroscopy Analysis

The circular dichroism (CD) spectra of the three casein products were recorded using a circular dichroism spectropolarimeter (Applied Photophysics Ltd., Leatherhead, Surrey, UK), as previously described [32]. Briefly, each casein sample was dissolved in phosphate-buffered saline (PBS; 0.01 mol/L, pH 7.4; Solarbio, Beijing, China) to a final concentration of 0.4 mg/mL. The sample solutions were transferred into a 1 mm path- length quartz cuvette and scanned under a continuous nitrogen purge at a constant rate of 200 nm/min over the wavelength range of 190–400 nm. Three replicate scans were collected for each sample.

The corresponding PBS spectrum was recorded under identical conditions and subtracted from the sample spectra as the background signal [33]. The resulting molar ellipticity data were analyzed using the BeStSel web server (version 1.3; http://bestsel.elte.hu). Data were uploaded in text format, with the input unit set to Measured ellipticity (mdeg), and the protein concentration, number of residues, and path length were entered. In the calculate the secondary structure module, the wavelength range was set to 190–250 nm, and the relative contents of secondary structure components were determined from the fitting results. Helix corresponds to α-helix; the sum of Parallel and Antiparallel corresponds to β-sheet; Beta-Turn corresponds to β-turn; and Rndm. Coil corresponds to random coil.

### 2.5. Microstructural Characterization

The microstructures of the three casein products were characterized by scanning electron microscopy (SEM; Carl Zeiss AG, Oberkochen, Germany) [34]. Samples were mounted on SEM stubs using conductive double-sided adhesive tape, sputter-coated with gold (Servicebio Technology Co., Ltd., Wuhan, China), and examined at an accelerating voltage of 2 kV and magnifications of 200×, 500×, and 10,000×.

### 2.6. Determination of Solubility

All reagents used in Section 2.6, Section 2.7 and Section 2.8 were of analytical grade and purchased from local commercial suppliers (Lanzhou, China), unless otherwise stated. The solubility (S) of casein was determined according to the standardized procedure for food protein solubility established by Morr et al. [35]. The protein contents of the supernatant and the total dispersion were determined using the Kjeldahl method. Briefly, 0.5 g of casein sample was accurately weighed and dispersed in 50 mL of deionized water. The dispersion was stirred at room temperature for 30 min, after which its pH was adjusted to 4.6 using 1 mol/L HCl with a pH meter (Shanghai INESA Scientific Instrument Co., Ltd., Shanghai, China). An aliquot of the dispersion was collected for determination of the total protein content. The remaining dispersion was centrifuged at 3000 rpm for 15 min using a centrifuge (Hunan Xiangyi Laboratory Instrument Development Co., Ltd., Changsha, China), and the resulting supernatant was collected for determination of the soluble protein content. The total nitrogen contents of the dispersion and supernatant were determined using the Kjeldahl method, and protein contents were calculated using the conversion factor of 6.38 as described in Section 2.1.

Solubility (%) = Protein content in the supernatant/Total protein content in the sample × 100(1)

### 2.7. Determination of Emulsifying Activity and Emulsion Stability

The emulsifying activity index (EAI) and emulsion stability index (ESI) of the three casein products were determined by a turbidimetric method, as described previously [36]. Briefly, 6.0 mg of each casein sample was dissolved in Tris–HCl buffer containing 0.5 mol/L NaCl and diluted to 6 mL to obtain a 0.1% (*w*/*v*) protein solution. Peanut oil (2 mL) was then added, and the mixture was homogenized at 12,000 rpm for 1 min using a high-speed homogenizer (IKA-Werke GmbH & Co. KG, Staufen, Germany) to form a primary emulsion. The final emulsion volume was 8 mL, corresponding to an oil-volume fraction of 0.25.

Immediately after homogenization, 50 μL of emulsion was withdrawn from the bottom of the sample and diluted approximately 100-fold with 5 mL of 0.1% (w/v) sodium dodecyl sulfate (SDS) solution. After rapid vortexing using a vortex mixer (Kylin-Bell Lab Instruments, Haimen, China), the absorbance was measured at 500 nm using UV–Vis spectrophotometer (Shimadzu Corporation, Kyoto, Japan) with0.1% SDS solution as the blank and recorded as A_0_. The emulsion was then held at room temperature for 10 min, after which a second 50 μL aliquot was collected from the same position and analyzed under identical conditions. The resulting absorbance was recorded as A_10_. The EAI and ESI were calculated using the following equations [37]:

(2) $$EAI\;\left(m^{2}/g\right)=\frac{2\times 2.303\times A_{0}\times N}{\rho \times w\times 10,000}\approx 184.24\times A_{0}$$

(3) $$ESI\;\left(min\right)=\frac{A_{0}\times 10}{A_{0}-A_{10}}$$

where N is the dilution factor, ρ is the oil-phase volume fraction before emulsification, and w is the protein mass concentration in the aqueous phase before emulsification.

### 2.8. Determination of Surface Hydrophobicity

Surface hydrophobicity ($H_{0}$) was determined using a hydrophobic fluorescent probe method with 1-anilinonaphthalene-8-sulfonic acid (ANS), according to a previously reported procedure [38]. Each casein product was dissolved in Tris-HCl buffer containing 0.5 mol/L NaCl to obtain protein concentrations of 0.005%, 0.01%, 0.02%, 0.05%, and 0.1% (*w*/*v*). Subsequently, 20 μL of 8.0 mmol/L ANS solution was added to 4 mL of the protein solution. The mixture was incubated at room temperature in the dark for 5 min.

The fluorescence intensity was measured using a fluorescence spectrophotometer (Hitachi High-Tech Corporation, Tokyo, Japan) at an excitation wavelength of 390 nm and an emission wavelength of 470 nm, with both the excitation and emission slit widths set to 5 nm. The fluorescence intensity was plotted against the protein concentration, and the initial slope of the linear regression was defined as the surface hydrophobicity index ($H_{0}$).

### 2.9. Data Analysis

All measurements, except for FTIR and CD, were conducted with three independent biological replicates, each independently prepared from a separate batch of raw material. The results are expressed as mean ± standard deviation. Statistical analysis was performed using one-way analysis of variance (ANOVA) with SPSS software (version 18.0; SPSS Inc., Chicago, IL, USA), followed by Tukey’s honestly significant difference (HSD) test for post hoc multiple comparison. Differences were considered statistically significant at *p* < 0.05. In the figures, “*” indicates *p* < 0.05, and “ns” indicates no significant difference. FTIR and CD spectra were obtained from single measurements and are presented as descriptive estimates without statistical comparison.

## 3. Results

### 3.1. Physicochemical Properties of the Three Casein Variants

The physicochemical properties of the three casein samples are summarized in Table 1. Imported casein and fresh yak milk casein showed similar protein contents of 96.96% and 96.85%, respectively, both higher than that of Qula-derived casein (96.43%), and all three samples met the national standard requirement of ≥ 90.0% protein for casein. The fat content was lowest in imported casein, followed by fresh yak milk casein, and highest in Qula-derived casein, which may be related to residual milk fat in the Qula raw material [39]. Moisture content varied little among the groups, ranging from 4.6% to 4.8%, well below the Chinese national standard limit of ≤12.0%. The ash content of Qula-derived casein (1.8%) was slightly higher than that of Imported casein and fresh yak milk casein, possibly due to minerals introduced during traditional processing. In terms of color and appearance, imported casein was a uniform pale-yellow powder, while fresh yak milk casein and Qula-derived casein exhibited slightly darker shades, likely resulting from Maillard reactions during processing. Overall, all three casein samples complied with the quality requirements of the Chinese national standard GB 31638-2016 (National Food Safety Standard—Casein) [19], indicating their suitability for subsequent structural and functional analyses.

### 3.2. Differential Proteomic Analysis

**Figure 1:**
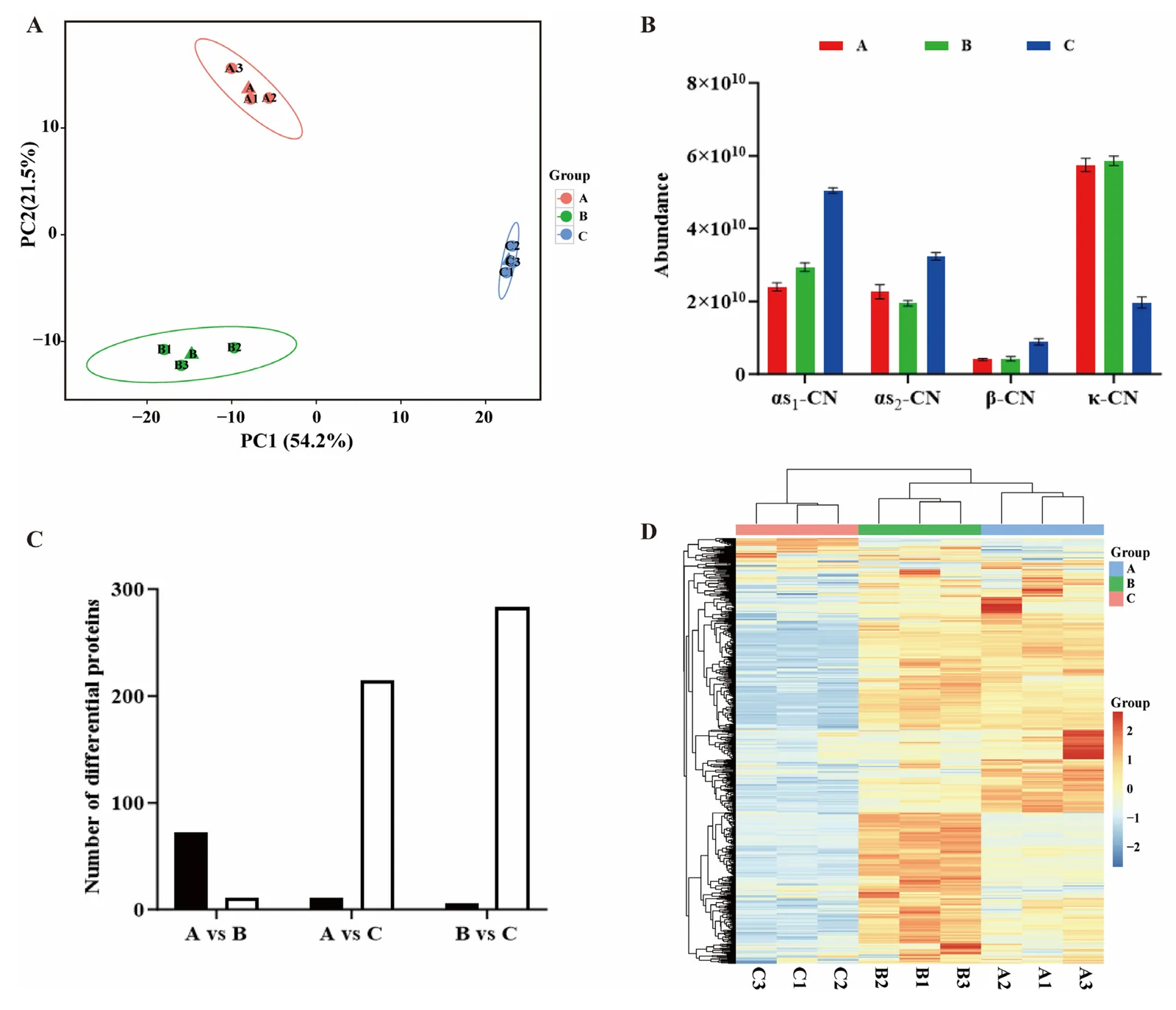
Proteomic analysis of the three casein samples. Sample A, imported casein; Sample B, fresh yak milk casein; Sample C, Qula-derived casein. (**A**) Principal component analysis (PCA) score plot. The numbers 1–3 indicate three biological replicates, and the unnumbered point represents the group mean. Ellipses indicate the distribution range of each sample group. (**B**) Relative abundances of the four casein subtypes. (**C**) Numbers of upregulated and downregulated proteins in the three pairwise comparison groups; filled squares indicate upregulated proteins, whereas open squares indicate downregulated proteins. (**D**) Heatmap of the abundance of all identified proteins. Each column represents one sample, and each row represents one protein. Different colors indicate different protein abundance levels, with darker and lighter colors representing higher and lower abundances, respectively.

Label-free quantitative proteomic analysis was performed on the three casein samples, with three biological replicates per group. A total of 563 quantifiable proteins were identified. Principal component analysis (PCA) revealed that the first principal component (PC1) and the second principal component (PC2) accounted for 54.2% and 21.5% of the total variance, respectively (Figure 1A). The three biological replicates within each group clustered closely together, indicating good intra-group reproducibility. Meanwhile, the three sample groups were clearly separated on the PCA plot, suggesting that their proteomic profiles differed significantly, thereby supporting the reliability of subsequent differential protein analyses [40]. Using FC ≥2 and a *p*-value <0.05, differentially abundant proteins (DAPs) were screened among the three comparison pairs. The abundance changes of four casein subtypes are presented in Figure 1B. The abundance of αs_1_-casein was higher in Qula-derived casein from yak milk than in imported casein; αs_2_-casein showed no marked differences among the three groups; β-casein abundance was higher in Qula-derived casein relative to imported casein and fresh yak milk casein; whereas κ-casein abundance was lower in Qula-derived casein than imported casein and fresh yak milk casein. The numbers of DAPs in each comparison are shown in Figure 1C. In the B vs. C comparison, a total of 289 DAPs were identified (6 up-regulated and 283 down-regulated), far exceeding those in the A vs. B and A vs. C comparisons, with the majority being down-regulated, indicating that the proteome of Qula-derived casein underwent more pronounced changes compared with that of fresh yak milk-derived casein. The heatmap of all quantified proteins across the three groups (Figure 1D) showed that the abundance of most proteins in Qula-derived casein was markedly lower than that in imported casein and fresh yak milk casein.

### 3.3. Comparison of Secondary Structural Features

**Figure 2:**
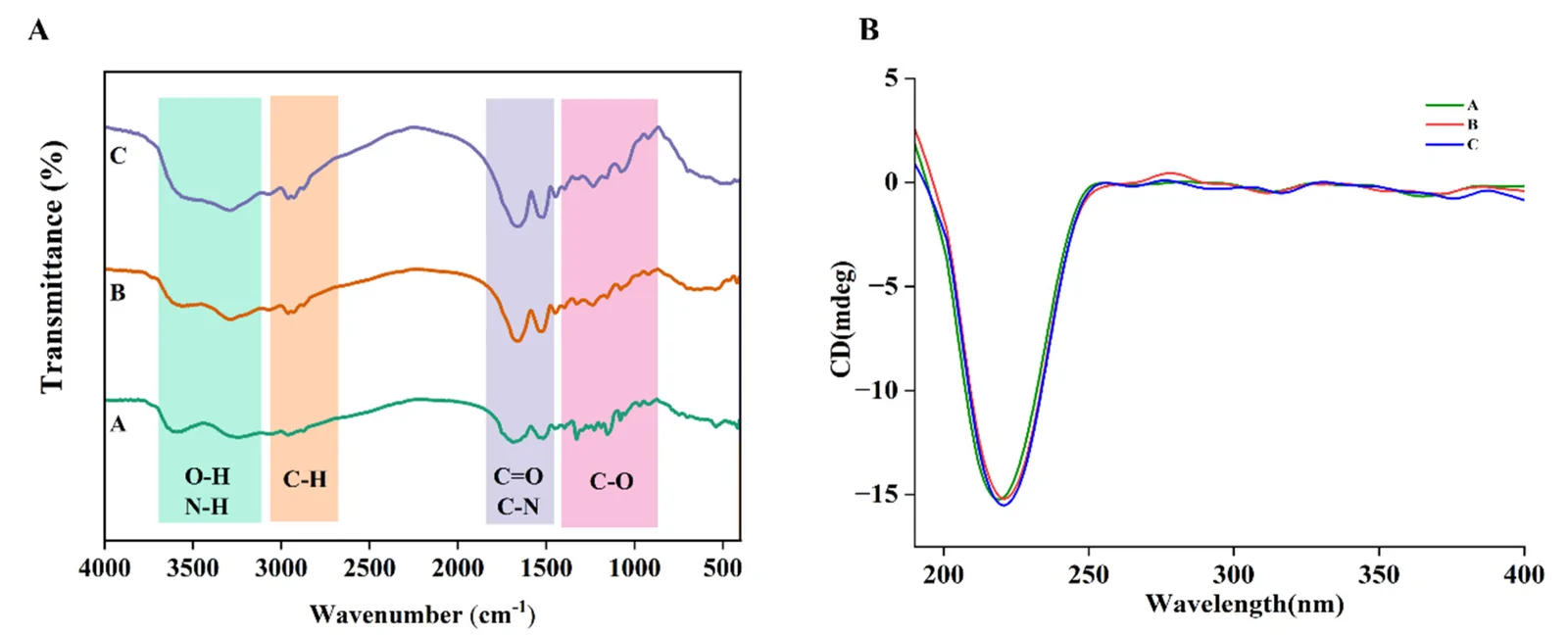
Secondary structure analysis of the three casein samples. Sample A, imported casein; Sample B, fresh yak milk casein; Sample C, Qula-derived casein. (**A**) FTIR spectra in the amide I region (4000–500 cm^−1^); (**B**) UV CD spectra (190–400 nm).

The structural characteristics of the three casein samples were analyzed by Fourier-transform infrared (FTIR) spectroscopy, and the spectra are shown in Figure 2A. In the region of 3700–3100 cm^−1^, corresponding to the overlapping O–H and N–H stretching vibrations, which reflect intermolecular hydrogen-bonding strength, imported casein exhibited a narrow peak with moderate intensity, indicating a well-ordered protein structure and stable hydrogen-bonding interactions. Fresh yak milk casein showed a slightly broadened peak, suggestive of mild structural alterations induced by acid precipitation processing [41]. In contrast, Qula-derived casein displayed a marked increase in peak width and intensity, implying that the natural fermentation, acid coagulation and prolonged sun-drying during Qula production may have contributed to protein aggregation and denaturation, with a substantially enhanced hydrogen-bonding network [42]. In the 3000–2800 cm^−1^ region, assigned to alkyl C–H stretching vibrations, the signal was weakest for imported casein, reflecting more thorough fat removal during industrial refining. Fresh yak milk casein and Qula-derived casein exhibited somewhat higher signals, which may be attributed to residual lipids in the raw material and alterations in the microenvironment of protein side chains [43]. In the amide I and amide II regions (1700–1500 cm^−1^), characteristic of protein secondary structure, imported casein showed a typical peak position and sharp shape, indicating intact secondary structure. Fresh yak milk casein exhibited a slight downward shift in peak position, suggesting minor structural adjustments due to acid precipitation, whereas Qula-derived casein showed the most pronounced shift, consistent with a higher degree of protein denaturation associated with traditional processing. In the 1300–1000 cm^−1^ region, associated with C–O and phosphate group vibrations, Qula-derived casein exhibited stronger absorption, likely related to the formation of Maillard reaction products and increased exposure of phosphate groups during processing.

The secondary structures of the three casein samples were further analyzed by circular dichroism (CD) spectroscopy, and the spectra are presented in Figure 2B. Sample A and Sample B displayed a typical positive peak at 192 nm and two negative peaks at 208 and 222 nm, indicative of intact α-helical structure. In contrast, the CD spectrum of Qula-derived casein showed alterations: the intensity of the positive peak at 192 nm decreased, while the negative peaks at 208 and 222 nm became shallower with a slight red shift, suggesting a reduction in α-helix content and an increase in random coil content, consistent with pronounced unfolding of the protein. The secondary structure contents of the three casein samples, determined from the CD spectra, are summarized in Table 2. Imported casein exhibited α-helix, β-sheet, random coil and β-turn contents of 10.2%, 32.5%, 31.5% and 25.8%, respectively. Fresh yak milk casein showed values of 8.5%, 30.1%, 34.6% and 26.8%, while Qula-derived casein had 5.8%, 26.4%, 42.3% and 25.5%, respectively. The β-turn content varied little among the three groups. Compared with fresh yak milk casein, Qula-derived casein displayed a 31.8% decrease in α-helix content, a 12.3% decrease in β-sheet content, and a 22.3% increase in random coil content. These data indicate that acid precipitation had only a limited effect on the secondary structure of casein, whereas the natural fermentation, acid coagulation and prolonged sun-drying involved in the traditional processing of Qula induced substantial unfolding of the protein, with a transition from ordered α-helix and β-sheet structures towards random coil. This finding is consistent with the FTIR spectroscopic analysis, and together, both techniques support the interpretation that thermal and traditional processing treatments promote the unfolding of casein structure [44].

### 3.4. Comparative Analysis of Surface Morphologies

**Figure 3:**
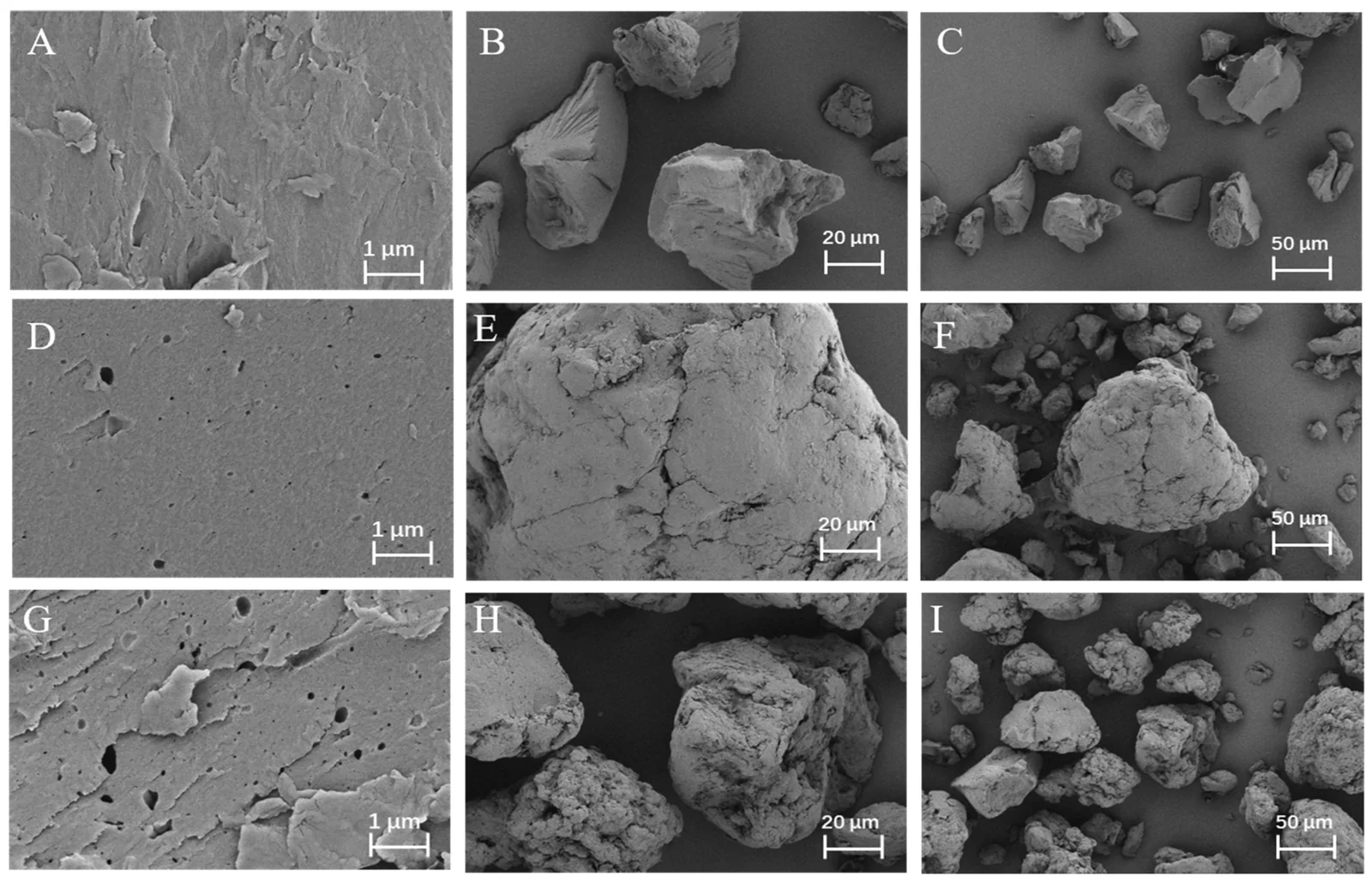
Scanning electron microscopy (SEM) images of the three casein samples at varying magnifications. Sample A, imported casein; Sample B, fresh yak milk casein; Sample C, Qula-derived casein. (**A**–**C**) Sample A, (**D**–**F**) sample B, and (**G**–**I**) sample C. Scale bars represent 10 μm, 20 μm, 50 μm.

The microstructures of the three casein samples were examined by scanning electron microscopy (SEM), and the micrographs are presented in Figure 3. Imported casein exhibited a relatively regular granular morphology, with uniformly sized particles, a fairly smooth surface, and good dispersibility. Fresh yak milk casein showed a particle morphology similar to that of imported casein; however, some particles appeared slightly rougher on the surface, with a few irregular fragments at the edges, which may be associated with mild structural changes induced by acid precipitation processing. In contrast, Qula-derived casein displayed a markedly altered morphology, transitioning from regular granules to a dense, blocky aggregated state, with increased particle size, rough surfaces, and evident adhesion and aggregation among particles. This pronounced change is likely attributable to the natural fermentation, acid coagulation and prolonged sun-drying involved in the traditional processing of Qula, which may have induced heat-mediated protein denaturation and exposure of hydrophobic groups, ultimately leading to irreversible intermolecular aggregation and the formation of a compact block-like network structure.

### 3.5. Comparative Analysis of Functional Properties

**Figure 4:**
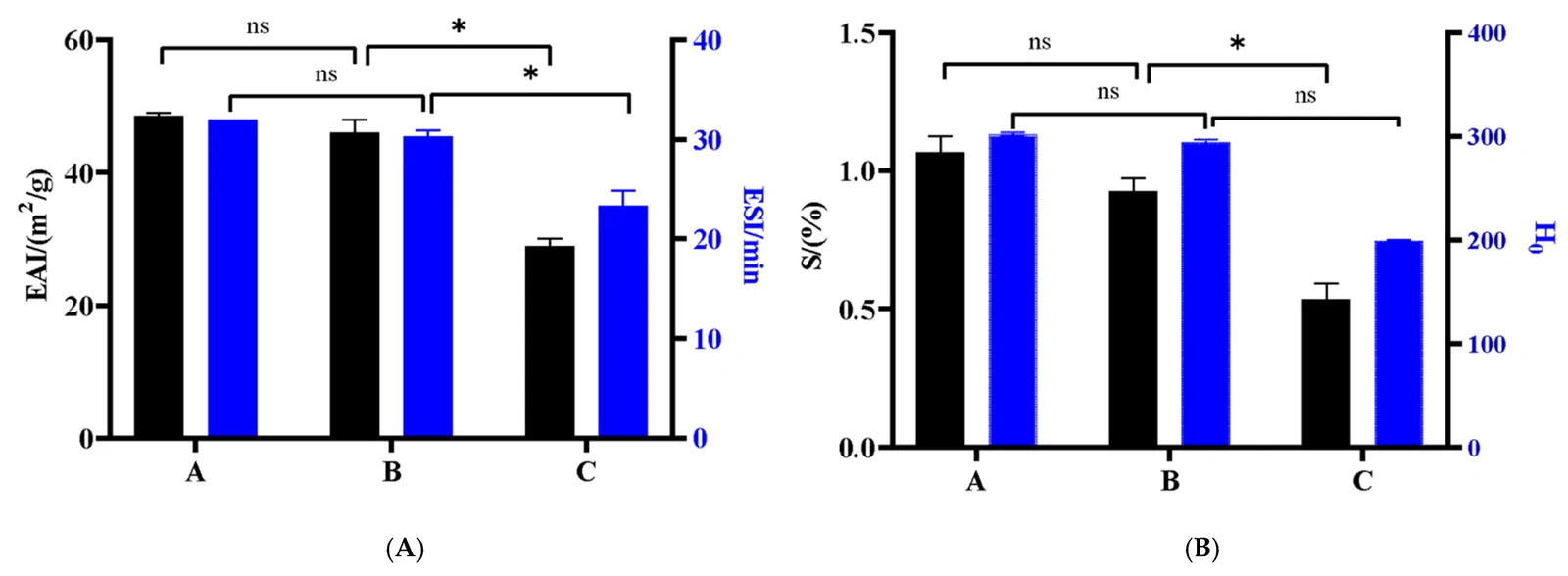
Functional properties of the three casein samples. Note: Sample A, imported casein; Sample B, fresh yak milk casein; Sample C, Qula-derived casein. “*” indicates *p* < 0.05, and “ns” indicates no significant difference. (**A**) Emulsifying activity and emulsion stability. (**B**) Solubility and surface hydrophobicity.

The emulsifying activity index (EAI) and emulsion stability index (ESI) of the three casein samples are presented in Figure 4A. The EAI values for imported casein, fresh yak milk casein, and Qula-derived casein were 48.5 m^2^/g, 46.3 m^2^/g, and 29.3 m^2^/g, respectively, while the corresponding ESI values were 32, 31, and 23 min. No significant differences in either EAI or ESI were observed between imported casein and fresh yak milk casein. However, Qula-derived casein exhibited significantly lower EAI and ESI than fresh yak milk casein, with reductions of 36.7% and 25.8%, respectively. The emulsifying performance of proteins is closely related to molecular flexibility, surface hydrophobicity, and aggregation state [45]. Imported casein and fresh yak milk casein retained relatively intact structures and good molecular chain flexibility, allowing rapid diffusion to the oil–water interface and formation of stable interfacial films; consequently, their emulsifying properties were comparable. In contrast, Qula-derived casein, which underwent natural fermentation, acid coagulation and prolonged sun-drying during Qula processing, exhibited substantial protein aggregation and unfolding. The excessive aggregation may have reduced molecular flexibility, thereby impairing rapid spreading and rearrangement at the oil–water interface and resulting in incomplete interfacial films, thus possibly decreasing both EAI and ESI. This finding is consistent with the observed changes in solubility and microstructure.

The solubility (S) at pH 4.6 and surface hydrophobicity (H_0_) of the three casein samples are shown in Figure 4B. The S values for imported casein, fresh yak milk casein, and Qula-derived casein were 1.1%, 0.9%, and 0.6%, respectively, and the corresponding H_0_ values were 301, 293, and 199. Imported casein and fresh yak milk casein showed no significant differences in either S or H_0_. Qula-derived casein, however, exhibited significantly lower S and H_0_ than fresh yak milk casein, with reductions of 33.3% and 32.1%, respectively. Imported casein, with its intact structure and low intermolecular aggregation, maintained relatively high solubility at the isoelectric point. Fresh yak milk casein, prepared by a similar process, displayed solubility comparable to that of imported casein. Although Qula-derived casein shared the same raw material origin as fresh yak milk casein, the traditional Qula processing (natural fermentation, acid coagulation and prolonged sun-drying) may induced heat-mediated irreversible protein aggregation, forming a dense blocky structure that facilitated precipitation at the isoelectric point, resulting in significantly lower solubility [46]. This observation is consistent with the microstructural changes revealed by SEM, indicating that heat-induced protein aggregation may be a factor contributing to the decreased solubility of Qula-derived casein. Regarding surface hydrophobicity, imported casein and fresh yak milk casei, with relatively intact structures and moderately exposed hydrophobic regions, allowed effective ANS probe binding, and their H_0_ values did not differ significantly. Although Qula-derived casein underwent unfolding, the excessive aggregation induced by thermal processing encapsulated hydrophobic groups within the aggregates, preventing effective ANS probe access, and thus its surface hydrophobicity was significantly lower than that of fresh yak milk casein [47]. These results, in conjunction with the solubility and emulsifying data, collectively suggest that heat-induced excessive aggregation may be a major factor contributing to overall deterioration of functional properties in Qula-derived casein.

### 3.6. Exploratory Correlation Analysis

**Figure 5:**
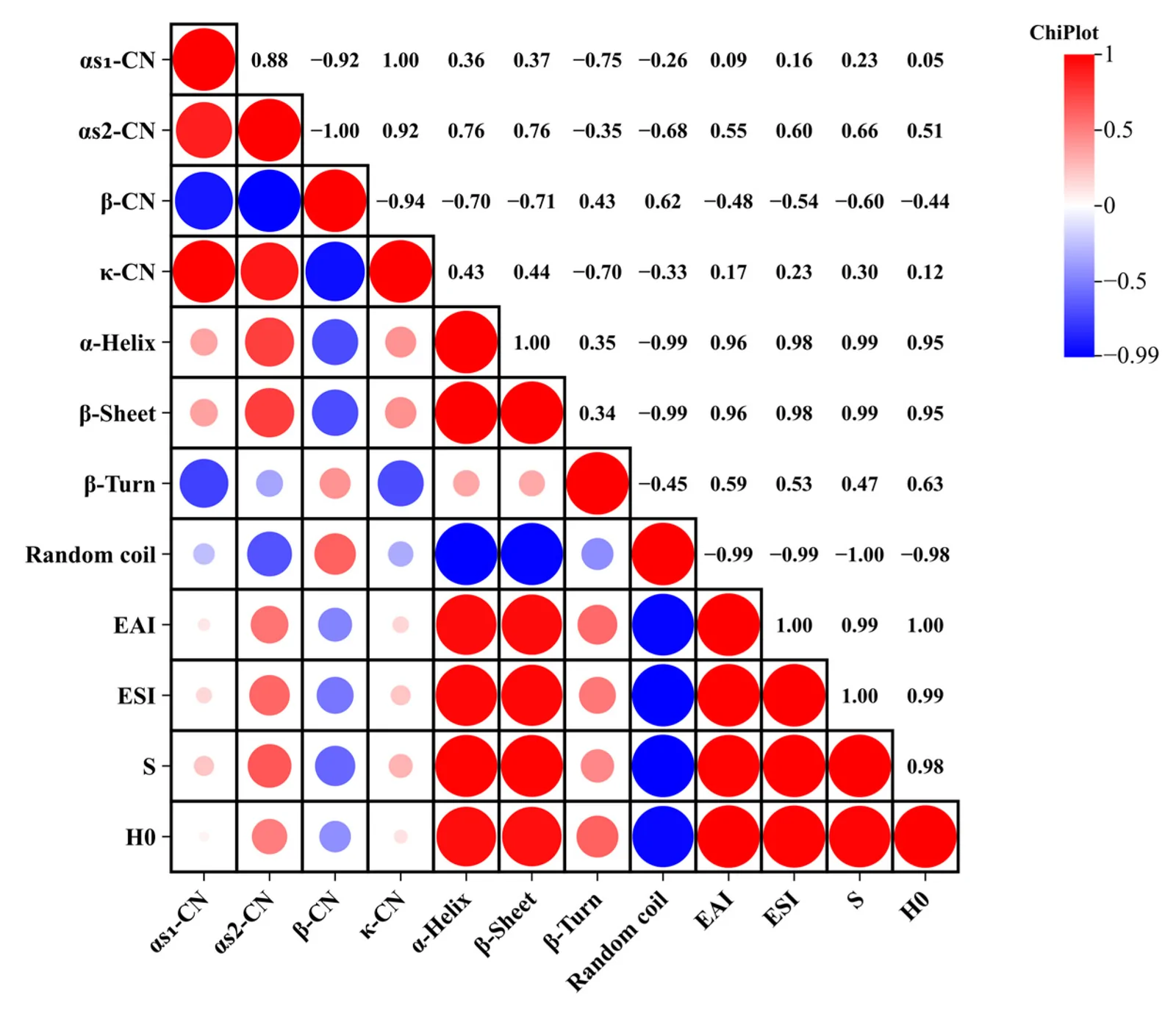
Correlation matrix of casein subtypes, secondary structure components, and functional properties. Red and blue colors indicate positive and negative correlations, respectively, and the intensity of the color corresponds to the correlation coefficient (r). The size of the circles is proportional to the absolute value of the correlation coefficient, with larger circles indicating stronger correlations. This figure is presented as an exploratory visualization. Correlations were calculated from the mean values of three sample groups (*n* = 3), and no significance testing or *p*-values are reported. The correlations should not be used to support mechanistic or causal relationships.

To explore the possible associations among casein subtype abundance, secondary structure content and functional properties, Pearson correlation analysis was performed based on the mean values of the three sample groups, and the results are presented in Figure 5. With respect to the correlations between casein subtypes and secondary structures, random coil content showed negative correlations with solubility (S) (*r* = −0.9996) and EAI (*r* = −0.987); descriptively, a higher degree of unfolding was associated with poorer solubility and emulsifying activity. Consistently, α-helix content showed positive correlations with EAI (*r* = 0.962) and S (*r* = 0.991). Among the functional properties, EAI showed a positive correlation with ESI (*r* = 0.998) and H_0_ (*r* = 0.999). Furthermore, κ-casein (*κ*-CN) exhibited a negative correlation with β-casein (β-CN) (*r* = −0.945) and a moderate negative correlation with random coil content (*r* = −0.326); tentatively, the reduction in κ-CN and the relative increase in β-CN during Qula processing were associated with structural unfolding.

## 4. Discussion

### 4.1. Variations in Proteomic Characteristics and Their Functional Implications

In this study, label-free quantitative proteomics was used to systematically characterize differences between Qula-derived casein and casein obtained from fresh yak milk. A total of 289 differentially abundant proteins (DAPs) were identified between fresh yak milk casein and Qula-derived casein, substantially more than those detected in the A vs. B and A vs. C comparisons. This pronounced difference suggests that natural fermentation and prolonged sun-drying during traditional Qula production may substantially reshape the casein-associated proteomic profile, with most DAPs exhibiting decreased abundance in Qula-derived casein relative to fresh yak milk casein. The four major casein subtypes appeared to respond differently to traditional Qula processing. In particular, the relative abundance of β-casein was higher in Qula-derived casein. This observation may be partly attributable to the comparatively high thermal stability of β-casein. Yoshino et al. [48] reported that β-casein released the lowest amount of non-protein nitrogen during heating, suggesting that it is less susceptible to heat-induced degradation than α_s- and κ-caseins. By contrast, the relative abundance of κ-casein was lower in Qula-derived casein. This finding is consistent with previous evidence showing that κ-casein releases the greatest amount of non-protein nitrogen upon heating. Moreover, six of its ten lysine residues are susceptible to lactose-mediated glycation, further indicating its vulnerability to heat-induced modification. Similarly, Lu et al. [49] demonstrated that κ-casein underwent more pronounced heat-induced modifications than the other casein subtypes. The relative abundance of α_s1-casein was also higher in Qula-derived casein. Nevertheless, changes in protein abundance do not necessarily reflect the extent of structural modification. Zeng et al. [50] reported that heating reduced the α-helical content and antigenicity of α_s1-casein, indicating that processing may alter its conformation and immunoreactivity even when its relative abundance is maintained or increased. In contrast, α_s2-casein remained relatively stable, suggesting a comparatively limited response to the processing conditions. Collectively, natural fermentation and prolonged sun-drying during traditional Qula production appeared to have subtype-specific effects on the major caseins: the relative abundances of α_s1- and β-casein increased, that of κ-casein decreased, and that of α_s2-casein remained relatively unchanged. These findings are broadly consistent with previously reported differences in the processing stability of casein subtypes. They further indicate that the overall reduction in protein abundance may mask distinct subtype-specific responses to traditional Qula processing.

### 4.2. Thermally Induced Structural Modifications of Casein and Their Implications for Functional Properties

The FTIR and CD analyses yielded consistent results, collectively indicating that processing appeared to substantially alter the secondary structure of casein. Compared with imported casein and fresh yak milk case, Qula-derived casein showed lower proportions of α-helical and β-sheet structures, together with an increased random-coil content, indicating a greater degree of conformational unfolding. This interpretation was further supported by the shift and broadening of the amide I band in the FTIR spectra, which suggested disruption of the hydrogen-bonding network and reorganization of the protein secondary structure. These findings are consistent with those of Acuña-Nelson et al. [51], who reported that ultra-high-temperature (UHT) treatment induced substantial secondary-structural changes in casein. However, the alterations observed in Qula-derived casein appeared to be more pronounced, possibly owing to its cumulative exposure to multiple processing steps and a relatively more extensive thermal history.

Scanning electron microscopy (SEM) provided direct visual evidence of processing-related differences in the microscopic morphology of the three casein samples. Imported casein exhibited relatively regular, well-dispersed particles with smooth surfaces, whereas fresh yak milk casein retained an essentially intact particulate structure but showed slightly roughened surfaces. In contrast, Qula-derived casein displayed a compact, block-like aggregated morphology, characterized by extensive particle adhesion and fusion. These morphological differences may reflect differences in the processing histories of the samples. Imported casein appeared to retain the most intact particulate structure. Fresh yak milk casein, produced through acid precipitation, exhibited only limited aggregation. Qula-derived casein, in contrast, underwent natural fermentation, heating, and prolonged sun-drying, which may have promoted extensive heat- and dehydration-induced protein aggregation. Similar findings were reported by Wang et al. [52] in their investigation of the effects of solar-drying conditions on Qula casein, where higher drying temperatures were associated with more extensive protein aggregation. Because this study compared final products rather than independently manipulating each processing step, the relative contribution of individual steps such as fermentation, heating, and sun-drying cannot be determined. Therefore, the mechanistic explanations above are presented as possible interpretations rather than proven causal effects.

### 4.3. Correlation Between Structural Modifications and Functional Properties

Protein functionality is closely associated with conformational state, intermolecular interactions, and aggregation behavior. In the present study, no significant differences were observed between imported casein and fresh yak milk casein in solubility, emulsifying activity index (EAI), emulsion stability index (ESI), or surface hydrophobicity (H_0_). These results indicate that the preparation procedure used for fresh yak milk-derived casein preserved the functional properties assessed in this study, resulting in performance comparable to that of the imported casein. By contrast, Qula-derived casein exhibited significantly lower values for all measured functional indices than fresh yak milk casein, suggesting that traditional Qula processing adversely affected casein functionality.

Casein solubility near its isoelectric point (pH 4.6) is strongly influenced by protein–protein interactions and aggregation. At this pH, the net charge of casein approaches zero, thereby weakening electrostatic repulsion and facilitating intermolecular association and precipitation. FTIR and CD analyses indicated that Qula-derived casein underwent substantial conformational rearrangement, as evidenced by decreases in α-helical and β-sheet structures and a concomitant increase in random-coil content. SEM observations further revealed compact, block-like morphology with extensive particle adhesion and fusion. Such extensive aggregation may restrict protein–water interactions and promote precipitation near the isoelectric point, thereby contributing to the significantly lower solubility of Qula-derived casein relative to fresh yak milk casein. This interpretation is broadly consistent with the findings of Liu et al. [53], who demonstrated that heat-induced conformational changes and hydrophobic association can promote protein aggregation and reduce solubility.

The emulsifying properties of proteins depend on several interrelated factors, including molecular flexibility, surface activity, hydrophobicity, solubility, and aggregation state. The relatively intact and less aggregated structure of fresh yak milk casein may have facilitated its diffusion to the oil–water interface, followed by molecular rearrangement and the formation of a cohesive interfacial film. Although partial unfolding can enhance interfacial activity by exposing hydrophobic regions, the extensive aggregation observed in Qula-derived casein likely reduced the number of soluble protein molecules available for adsorption and limited their conformational mobility at the interface. These effects may have impeded the rapid formation and rearrangement of the interfacial layer, resulting in significantly lower EAI and ESI values than those of fresh yak milk casein. Similarly, Hu et al. [54] reported source-dependent differences in the structural and functional properties of sodium caseinates, with less structurally disrupted samples generally exhibiting better emulsifying performance. Their findings support the association observed here between the aggregation state of casein and its emulsifying properties.

Surface hydrophobicity, as determined using the 8-anilino-1-naphthalenesulfonic acid (ANS) probe, reflects the accessibility of hydrophobic regions on the protein surface rather than the total number of hydrophobic groups within the molecule. Fresh yak milk casein retained a relatively intact and dispersed structure, allowing accessible hydrophobic regions to interact effectively with ANS. In Qula-derived casein, initial conformational unfolding may have exposed additional hydrophobic residues; however, subsequent intermolecular hydrophobic association and extensive aggregation may have buried some of these residues within the aggregates. The compact aggregated structure may also have sterically restricted probe access to the remaining hydrophobic regions. These effects could explain why Qula-derived casein exhibited lower apparent surface hydrophobicity than fresh yak milk casein despite showing evidence of conformational unfolding. A similar biphasic response was reported by Al-Wraikat et al. [55], who observed an initial increase followed by a decrease in the surface hydrophobicity of whey proteins during heating. This pattern suggests that moderate unfolding increases the accessibility of hydrophobic regions, whereas extensive aggregation subsequently reduces their accessibility to fluorescent probes.

Collectively, the impaired functional properties of Qula-derived casein appear to result from the combined effects of conformational rearrangement and extensive protein aggregation. The compact, block-like aggregates observed in Qula-derived casein may have weakened protein–water interactions and reduced solubility; decreased molecular mobility and the limited availability of soluble protein may have impaired adsorption and rearrangement at the oil–water interface, thereby possibly reducing emulsifying activity and emulsion stability; and the burial or steric shielding of hydrophobic regions within aggregates may have lowered their accessibility to the ANS probe. These findings provide a plausible structure–function explanation for the reduced solubility, emulsifying performance, and apparent surface hydrophobicity of Qula-derived casein following traditional processing. From the perspective of industrial practice, Qula, as a casein-rich material, already has a basis for industrial utilization. Industrial processes typically involve standardized raw material collection, controlled fermentation and drying, and spray drying, with quality control implemented in accordance with GB 31638–2016 which specifies limits for microbial contamination, pollutants, and mycotoxins. The mechanism of κ-casein loss and irreversible aggregation identified in this study may provide a basis for optimizing heat treatment intensity and drying time in these processes. Nevertheless, systematic evaluation under different pH, salt, and processing conditions is needed to further support the application relevance of Qula-derived casein.

## 5. Conclusions

In this study, the proteomic, structural, and functional characteristics of Qula-derived casein from yak milk were systematically compared with those of fresh yak milk casein, with imported casein being used as an industrial reference control. Proteomic analysis identified a total of 289 differentially abundant proteins (DAPs). In Qula-derived casein, the abundance of αs_1_-casein was higher than in imported casein, and β-casein was higher compared with both imported casein and fresh yak milk casein, whereas κ-casein was lower than in imported casein and fresh yak milk casein. Compared with fresh yak milk casein, Qula-derived casein exhibited a 31.8% decrease in α-helix content, a 12.3% decrease in β-sheet content, and a 22.3% increase in random coil, with a microstructure transition from loose, porous particles to dense, blocky aggregates. These structural changes were associated with reduced solubility at the isoelectric point, impaired interfacial film formation, and encapsulation of surface hydrophobic groups. Correspondingly, EAI, ESI, solubility at pH 4.6, and H_0_ decreased to 29.3 m^2^/g, 23 min, 0.6% and 199, respectively reductions of 36.7%, 25.8%, 33.3% and 32.1% compared with fresh yak milk casein. Imported casein and Qula-derived casein share a casein-dominant profile, but Qula-derived casein has higher β-casein, lower κ-casein, and greater unfolding and aggregation, with lower solubility, emulsifying activity, and surface hydrophobicity than imported casein and fresh yak milk casein.

These results suggest an association between functional deterioration and κ-casein loss and irreversible aggregation. However, because this study compared final products rather than independently manipulating each processing step, a primary causal mechanism cannot be established. The functionality of the final products could potentially be improved by optimizing drying and heat treatment to reduce κ-casein loss and inhibit excessive aggregation. For already deteriorated Qula-derived casein, use in food systems with lower emulsifying requirements or post-modification utilization may be feasible. The transformation of Qula from an artisanal to an industrial product can be supported by standardized raw material collection, controlled fermentation and drying, spray drying, and quality control in accordance with GB 31638–2016; the observed association between κ-casein loss and irreversible aggregation provides a basis for optimizing heat treatment intensity and drying time. Further studies under different pH, salt, and processing conditions are needed to confirm its application potential. Using Qula as a model, this study provides insight into the proteomic basis and molecular mechanisms of unfolding and aggregation, and suggests possible associations between casein subtype alterations and functional properties.

## Figures and Tables

**Table 1:** Physicochemical parameters of the three casein samples.

Parameter/%	Sample A	Sample B	Sample C
Protein	96.96 ± 0.4 ^a^	96.85 ± 0.5 ^a^	96.43 ± 0.6 ^b^
Fat	1.1 ± 0.1 ^a^	1.2 ± 0.1 ^a^	1.4 ± 0.2 ^b^
Moisture	4.6 ± 0.2 ^a^	4.7 ± 0.1 ^a^	4.8 ± 0.2 ^a^
Ash	1.5 ± 0.1 ^a^	1.6 ± 0.1 ^a^	1.8 ± 0.2 ^a^

Note: Sample A, imported casein; Sample B, fresh yak milk casein; Sample C, Qula-derived casein. Different letters indicate statistically significant differences between samples (*p* < 0.05), while the same letters indicate no significant difference (*p* > 0.05). Protein is expressed on a dry basis; all other parameters are expressed on a wet basis.

**Table 2:** Relative contents of secondary structure elements (α-helix, β-sheet, β-turn, and random coil) in the three casein samples.

Secondary Structure Type	Sample A	Sample B	Sample C
α-Helix	10.2	8.5	5.8
β-Sheet	32.5	30.1	26.4
β-Turn	25.8	26.8	25.5
Random coil	31.5	34.6	42.3

Note: Sample A, imported casein; Sample B, fresh yak milk casein; Sample C, Qula-derived casein.

## Data Availability

All data generated or analyzed during this study are included in this article, except for the raw proteomics data, which has been deposited in the ProteomeXchange Consortium via the iProX partner repository under accession number PXD083972. The dataset is available at https://www.iprox.cn/page/PSV023.html?url=1789089999930MXrs (accessed on 10 September 2026, password: fT50). Further inquiries can be directed to the corresponding authors.

## Supplementary Materials

The following supporting information can be downloaded at: https://www.mdpi.com/article/10.3390/foods15203669/s1, Figure S1: SDS-PAGE profiles of Sample A (imported casein), Sample B (fresh yak milk casein), and Sample C (Qula-derived casein). The main bands corresponding to αs1/αs2-casein, β-casein, and κ-casein are indicated.
