# Supplementary material for: Proteomics-Based Analysis of Structural Changes and Functional Properties of Casein Derived from Qula
Source: Foods. 2026 Oct 15;15(20):3669. doi: 10.3390/foods15203669 (PMC13703591; doi:10.3390/foods15203669)
Supplement: Supplementary file 1 [file foods-15-03669-s001.zip › foods-4573233-supplementary.pdf]

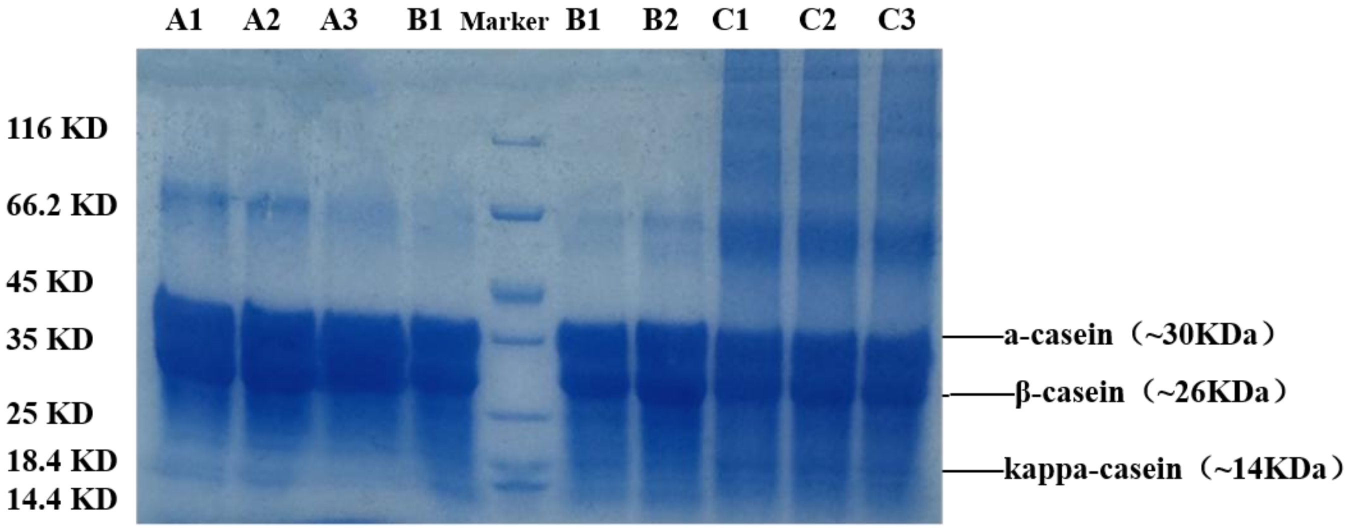

Figure S1. SDS-PAGE profiles of Sample A (imported casein), Sample B (fresh yak milk casein), and Sample C (Qula-derived casein). The main bands corresponding to αs1/αs2-casein, β-casein, and κ-casein are indicated.
